# Editorial for the Special Issue on Miniature Optoelectronic Resonators and Oscillators

**DOI:** 10.3390/mi13111928

**Published:** 2022-11-08

**Authors:** Patrice Salzenstein

**Affiliations:** Centre National de la Recherche Scientifique (CNRS), Franche-Comté Electronique Mécanique Thermique Optique Sciences et Technologies (FEMTO-ST) Institute, Université Bourgogne Franche-Comté (UBFC), 25000 Besançon, France; patrice.salzenstein@femto-st.fr

The idea of developing oscillators, which can potentially replace electric oscillators such as those based on quartz, is interesting. Since their introduction almost thirty years ago, optoelectronic oscillators (OEO) have been a family of potential candidates whose performance can be expected to compete with more conventional oscillators, and even provide solutions which are less sensitive to external parameters. After years of research, considerable progress has been made in the wake of the pioneers in this field. Creating miniature OEOs is a constantly developing field. There are many challenges that researchers must overcome to achieve their goals. The miniature OEOs for the most efficient applications must satisfy conditions such as staying in very low phase noise levels, while occupying a low volume. For miniature optoelectronic resonators and OEOs intended to be integrated, it is fundamental to design and manufacture relatively robust structures on chips while ensuring high quality coefficients and consequent yields. Accordingly, this Special Issue seeks to showcase research papers, short communications, and review articles that focus just as much on the efforts made at the level of the design as the technological obstacles that it has been necessary to remove, but also on the state-of-the-art current performances of miniature OEOs.

Out of the five articles published in this volume of this Special Issue, four are original research papers and one is a review article. Four papers were submitted from China, and one paper was contributed from France.

In this Special Issue on miniature optoelectronic resonators and oscillators, we include five papers, covering different aspects related to Fano resonance [1], a tunable metamaterial absorber [2], uncertainty evaluation on the signal delivered by an OEO [3], and a high quality factor of a filter with an ultra-narrow linewidth [4], as well as a review on high-power all-solid-state single-frequency continuous-wave lasers [5].

Specifically, Chen describes the nanoresonator enhancement of majorana-fermion-induced slow light in superconducting iron chains [1]. Xu et al. present a dynamically switchable polarization-independent triple-band perfect metamaterial absorber using a phase-change material in the mid-infrared (MIR) region [2]. Salzenstein and Pavlyuchenko report on uncertainty evaluation on a 10.52 GHz (5 dBm) optoelectronic oscillator regarding its phase noise performance [3]. Xu et al. demonstrate an all-dielectric color filter with ultra-narrow linewidth [4]. The review article of Peng et al. in this issue examines high-power all-solid-state single-frequency continuous-wave lasers [5].

We hope that this Special Issue of *Micromachines* will offer readers a good overview of the current state of the art in this fast-growing area of research as well as an introduction to some of the newest techniques developed in the field.

## References

[1] Chen H. (2021). Nanoresonator Enhancement of Majorana-Fermion-Induced Slow Light in Superconducting Iron Chains. Micromachines.

[2] Xu D., Cui F., Zheng G. (2021). Dynamically Switchable Polarization-Independent Triple-Band Perfect Metamaterial Absorber Using a Phase-Change Material in the Mid-Infrared (MIR) Region. Micromachines.

[3] Salzenstein P., Pavlyuchenko E. (2021). Uncertainty Evaluation on a 10.52 GHz (5 dBm) Optoelectronic Oscillator Phase Noise Performance. Micromachines.

[4] Xu K., Meng Y., Chen S., Li Y., Wu Z., Jin S. (2021). All-Dielectric Color Filter with Ultra-Narrowed Linewidth. Micromachines.

[5] Peng W., Jin P., Li F., Su J., Lu H., Peng K. (2021). A Review of the High-Power All-Solid-State Single-Frequency Continuous-Wave Laser. Micromachines.

